# Sex-Specific Effects of Early Life Stress on Brain Mitochondrial Function, Monoamine Levels and Neuroinflammation

**DOI:** 10.3390/brainsci10070447

**Published:** 2020-07-14

**Authors:** Héctor González-Pardo, Jorge L. Arias, Eneritz Gómez-Lázaro, Isabel López Taboada, Nélida M. Conejo

**Affiliations:** 1 Laboratory of Neuroscience, Department of Psychology, Institute of Neuroscience of the Principality of Asturias (INEUROPA), University of Oviedo, Plaza Feijóo, s/n E-33003 Oviedo, Spain; hgpardo@uniovi.es (H.G.-P.); jarias@uniovi.es (J.L.A.); UO238998@uniovi.es (I.L.T.); 2 Department of Basic Psychological Processes and their Development, Basque Country University, Avda. Tolosa 70, s/n E-20018 San Sebastian, Spain; eneritz.gomez@ehu.eus

**Keywords:** maternal separation, sex, cytochrome oxidase, cytokine, monoamine, prefrontal cortex, hippocampus, striatum

## Abstract

Sex differences have been reported in the susceptibility to early life stress and its neurobiological correlates in humans and experimental animals. However, most of the current research with animal models of early stress has been performed mainly in males. In the present study, prolonged maternal separation (MS) paradigm was applied as an animal model to resemble the effects of adverse early experiences in male and female rats. Regional brain mitochondrial function, monoaminergic activity, and neuroinflammation were evaluated as adults. Mitochondrial energy metabolism was greatly decreased in MS females as compared with MS males in the prefrontal cortex, dorsal hippocampus, and the nucleus accumbens shell. In addition, MS males had lower serotonin levels and increased serotonin turnover in the prefrontal cortex and the hippocampus. However, MS females showed increased dopamine turnover in the prefrontal cortex and increased norepinephrine turnover in the striatum, but decreased dopamine turnover in the hippocampus. Sex differences were also found for pro-inflammatory cytokine levels, with increased levels of TNF-α and IL-6 in the prefrontal cortex and hippocampus of MS males, and increased IL-6 levels in the striatum of MS females. These results evidence the complex sex- and brain region-specific long-term consequences of early life stress.

## 1. Introduction

There is compelling evidence supporting the hypothesis that early exposure to adverse or stressful life experiences (ELS) like childhood abuse or neglect increases the vulnerability to develop during adulthood the most prevalent mental disorders like depression, anxiety, and substance use disorders, but also common physical health problems like obesity, cardiovascular diseases, cancer, diabetes, neurodegenerative diseases, among others [1,2,3,4]. Clinical and animal studies have suggested that the quality of parental and/or maternal care during early postnatal life has a lasting and profound effect on brain development and adult behavior [5,6,7]. In addition, ELS affects not only brain function and behavior, but it can lead to dysregulated neurochemical, neuroendocrine, and immune responses during adulthood. In particular, childhood abuse and parental neglect increase adult inflammatory responses and alters neuroendocrine stress responses mediated by the hypothalamic-pituitary-adrenal (HPA) axis [3,6,8,9,10,11]. Moreover, studies using animal models of ELS like prolonged maternal separation (MS) in rodents (in which the offspring are daily separated from their mothers before weaning) have been shown to alter adult brain monoamine levels in several brain regions related with abnormal behavior, like dopamine, serotonin, and norepinephrine [12,13,14,15].

Recently, it has been proposed that mitochondria have a pivotal role as both key mediators and targets of the physiological stress response, as the only mammalian cell organelle responsible for energy production required to adapt and respond to environmental stressors [16,17,18,19,20]. ELS can modulate mitochondrial function because stress response mediators like cortisol and catecholamines are both synthesized and metabolized by mitochondria [16] but also the stress response essentially involves energy production and increased availability of energy substrates like glucose in the brain, one of main energy-demanding organs in the body [21]. In addition, prolonged exposure to psychosocial stress has been linked to mitochondrial dysfunction by increased oxidative stress that can lead to increased neuroinflammatory response mediated by cytokine release and pro-inflammatory gene expression, together with increased glucocorticoid production by mitochondria [22]. On the other hand, chronic psychological stress and ELS are mainly associated with altered mitochondrial metabolic capacity mediated by respiratory chain enzymes of brain cells in human and animal studies [17,23,24,25]. In particular, childhood adversity and maltreatment have been linked to altered mitochondrial function, including increased oxidative stress and decreased mitochondrial respiratory chain activity [17,20].

However, the main limitation of animal studies about the effects of early psychological stress on mitochondrial function, but also on its neuroendocrine and neuroimmune correlates, is the almost exclusive use of male animals [16,17]. In this regard, significant sex differences have been reported in mitochondrial function and their responses to stressors [26,27]. It is known that mitochondria are sensitive to sex hormones and that these hormones regulate mitochondrial function in brain tissue [28]. Moreover, neuroendocrine and neuroimmune responses to psychological stress are also sexually dimorphic [29,30]. Lastly, there are a few studies reporting sex differences in rodents after prolonged MS or chronic stress in brain oxidative capacity, an index of mitochondrial function [31,32] brain cytokines [30,33], and brain monoamines [13]. Since estrogens seem to have significant anti-inflammatory and antioxidant effects, and brain mitochondrial energy metabolism is higher in female than in male rodents [26], it could be hypothesized that females would be less vulnerable to the effects of ELS.

Given the paucity of studies about sex differences in the long-term effects of ELS on brain mitochondrial function, neurochemistry, and neuroinflammation, the present study aimed to evaluate these effects after prolonged MS in male and female rats over the entire weaning period (PND 1-21, 4 h/day) [34,35,36,37,38,39]. Evaluation of brain mitochondrial function was performed by quantification of regional brain oxidative metabolic capacity using cytochrome c oxidase (CCO) quantitative histochemistry. CCO (also known as mitochondrial Complex IV, EC 1.9.3.1) is a mitochondrial enzyme complex involved in cellular respiration and energy metabolism because it is directly responsible for cellular oxygen consumption, as the final member of the electron transport chain in the mitochondria. Changes in brain CCO activity are directly coupled with changes in neuronal activity in order to meet its energy demands [40]. Quantitative CCO histochemistry in brain tissue has been applied in previous studies using several MS protocols in male or female rodents by our research group and others [32,34,35,41]. Brain oxidative capacity in selected regions of interest (prelimbic and infralimbic areas of the prefrontal cortex, cingulate cortex, dorsal and ventral hippocampus, and striatum) will be evaluated in adult (70-day-old) male and female rats after early prolonged MS as already described. Moreover, possible brain neuroinflammatory responses in these rats were assessed by the determination of cytokine mRNA expression in the selected brain regions (IL-6, TNF-alpha) by quantitative polymerase-chain-reaction (PCR). Lastly, monoaminergic activity in the same brain regions was quantified by high-performance liquid chromatography (HPLC) by determining the relative levels of dopamine, norepinephrine, and serotonin (5-HT), and their main metabolites: 3,4-dihydroxyphenylacetic acid (DOPAC), 3-methoxy-4-hydro-xyphenylglycol (MHPG) and 5-hydroxyindoleacetic acid (5-HIAA).

## 2. Materials and Methods

### 2.1. Animals and Maternal Separation Procedure

Adult pregnant female Wistar rats weighing 250 ± 30 g were obtained from the Production and Experimentation Animal Center (Institute of Biomedicine, University of Seville, Seville, Spain) and individually housed under controlled room temperature, humidity, and a 12 h daylight cycle (8:00–20:00 h). After delivery, litters were cross-fostered and distributed across dams in groups of 10–12 pups, with an equal male-to-female ratio. The day of birth was considered as postnatal day 0 (P0). The anogenital distance was used to differentiate males from females at this age. All experimental procedures and animal care were conducted according to the European Union Directive 2010/63/UE on care and use of animals for scientific purposes and the Spanish legislation on care and use of animals for experimentation (Royal Decree 53/2013). The experimental protocols used in this study were approved by the Ethics Committee of the University of Oviedo (Oviedo, Spain).

The litters were randomly distributed in two groups as previously described [35,36,38,39]: maternal separation (MS) group with daily separation of the whole litter in an incubator at 30 °C during 4 h from P1 through P21, and control group with unhandled litters during the same time period. After weaning on P21, male and female pups were removed and housed separately in groups of 4 to 5 animals per cage until adulthood (P90). A total of four experimental groups were obtained according to sex and rearing condition manipulation (MS or control) with 15 animals per group. Rats had tap water and food (Teklad global 14% protein rodent maintenance diet, Envigo, Barcelona, Spain) available ad libitum during this period.

### 2.2. CCO Quantitative Histochemistry

After decapitation of rats from the four groups at P90, brains from 10 animals were quickly frozen in isopentane at −80 °C and processed for quantitative cytochrome c oxidase (CCO) histochemistry following the original method by Gonzalez-Lima and Cada [42] as previously described [32,39,43]. In brief, CCO histochemistry was performed in serial 30-µm-thick coronal sections mounted in microscope slides. Brain tissue was lightly fixed in paraformaldehyde and sections were incubated at 37 °C in a cytochrome c (Sigma–Aldrich, Madrid, Spain) and diaminobenzidine (Sigma-Aldrich, Madrid, Spain) phosphate-buffered solution. The histochemical reaction was stopped with buffered formalin, and sections were dehydrated, and coverslipped. Brain homogenate sections of known CCO activity by spectrophotometry were used as staining standards. CCO activity was quantified by optical densitometry using a digital densitometry system (MCID Core, InterFocus Imaging Ltd., Linton, England). Twelve measurements at different rostro-caudal anatomical levels were done in the following brain regions according to the Paxinos and Watson atlas [44]: prelimbic (PL) area of the medial prefrontal cortex, the dorsal striatum (STR), the nucleus accumbens core (NAcC) and shell (NAcS) subdivisions, the hippocampal subfields of the dorsal (dCA1, dCA3, dDG) and ventral hippocampus (vCA1, vCA3).

Brains from five animals were directly dissected to isolate the prefrontal cortex, the hippocampus, and the striatum as previously described [45] and frozen in isopentane. Brain tissue from all subjects in each experimental group was batch processed because of the low amount of tissue containing each brain region in each rat, particularly for HPLC analysis. Next, brain tissue samples were processed for high-performance liquid chromatography (HPLC) and PCR analysis, as described in the following sections.

### 2.3. Determination of Brain Monoaminergic Activity

Monoaminergic activity was evaluated by assessing serotonin (5-HT), dopamine (DA), and noradrenaline (NA) levels in the prefrontal cortex, striatum, and hippocampus for each experimental group. Their respective metabolite levels, including 5-hydroxyindoleacetic acid (5-HIAA), 3,4-dihydroxyphenylacetic acid (DOPAC), and 3-methoxy-4-hydro-xyphenylglycol (MHPG) were also analyzed. Moreover, 5HIAA/5HT, DOPAC/DA, and MHPG/NA ratios were calculated.

These results were obtained via high-performance liquid chromatography (HPLC) using an Agilent 1200 LC system (Agilent Technologies, Madrid, Spain) equipped with a vacuum degasser, quaternary pump, cooled autosampler, thermostated column compartment, and fluorescence and variable wavelength detectors. The chromatographic separation was performed on a Poroshell 120 EC-C18 column (100 × 4.6 mm, 2.7 μm) protected by a cartridge guard column (Agilent Technologies, Madrid, Spain). The mobile phase consisted of 0.05% trifluoroacetic acid (solvent A) and acetonitrile (solvent B). The flow was maintained at a constant rate of 0.5 mL/min. The gradient elution program was as follows: from 0 to 8 min, 6% solvent B (*v*/*v*); from 8 to 15 min, 10% solvent B (*v*/*v*); from 15 to 22 min, 20% solvent B (*v*/*v*); and from 22 to 25 min, 2% solvent B (*v*/*v*). The column was maintained at 25 °C during the analysis, and samples were maintained at 4 °C in an autosampler unit. The effluent was monitored with the fluorescence detector at excitation wavelengths of 283 nm for DOPAC and 5-HIAA, 212 nm for NA, MHPG, and DA, and 229 nm for 5-HT. For all analyses, the emission wavelength was 320 nm. The total sample analysis time was 25 min. The mobile phase was prepared daily and filtered through a 0.22-μm Durapore filter (Millipore, Madrid, Spain). Prior to the sample preparation, the frozen tissues were weighed on AG204 analytical scales (Mettler Toledo, Columbus, OH, USA). The tissues were homogenized and deproteinized in a 60 μL homogenization solution (1% formic acid in acetonitrile) in a Bullet Blender homogenizer (Next Advance, New York, NY, USA; BBY24 M Bullet Blender Storm) and subsequently centrifuged for 15 min at 15,000× *g* and 4 °C (Beckman Coulter, Madrid, Spain; Microfuge 22R Centrifuge). The supernatants were dried for 30 min with compressed air to concentrate the samples and subsequently reconstituted with 30 μL of 0.05% trifluoroacetic acid. Given that it is impossible to filter such small volumes, the samples were centrifuged for 15 min at 15,000× *g* and 4 °C. Ultimately, 20 μL of each supernatant was injected into the HPLC system for analysis. Data processing was performed with the Agilent ChemStation software program (Agilent Technologies, Madrid, Spain); this program was used to quantify all compounds by comparing the areas under the peaks with the areas of the reference standards. All standards were purchased from Sigma–Aldrich (St. Louis, MO, USA) and dissolved in a stock 0.1 N hydrochloric acid solution. The calibration samples were prepared by adding appropriate amounts of standard working solutions to chromatographic grade water obtained from an ultrapure water system (Millipore, Madrid, Spain).

### 2.4. Real-Time PCR Analysis of mRNA Cytokine Expression

The total RNA of each structure was isolated using the NucleoSpin RNA Plus kit (Macherey Nagel, Germany). A UV spectrophotometric analysis was performed at 260 nm to determine the RNA concentrations, while the 260:280 absorbance ratio was utilized to assess the nucleic acid purity. (Synergy HT, BioTek Instruments, Inc., Winooski, VT, USA). The total RNA was then reverse-transcribed with the PrimeScript RT reagent kit (Takara Bio Inc., Madrid, Spain). The resulting cDNA levels were quantified by SYBR Green-based (SYBR^®^Premix Ex TaqTM, Takara Bio Inc., Madrid, Spain) real-time PCR, and the formation of PCR products was monitored using the 7500 Real-Time PCR System (Applied Biosystems, Madrid, Spain). The cDNA sequences were obtained from GenBank at the National Center for Biotechnology Information (www.ncbi.nlm.nih.gov). Glyceraldehyde-6-phosphate dehydrogenase (GAPDH) served as a housekeeping gene. The primer sequences were designed using Primer Express Software v3.0 (Applied Biosystems, Madrid, Spain), and obtained from Applied Biosystems. Prime specificity was verified by melt curve analysis. The relative gene expression was determined using the 2−ΔΔt method [46]. Primer sequences used for PCR were as follows (primer sequence direction is 5′….3′): IL-6, forward: CCACCAGGAACGAAAGTCAAC, reverse: CTTGCGGAGAGAAACTTCATAGC; TNF-α, forward: GCCACCGGCAAGGATTC, reverse: TCGACATTCCGGGATC CA; GAPDH, forward: GCTCTCTGCTCCTCCCTGTTC, reverse: GAGGCT GGCACTGCACAA.

### 2.5. Statistical Analysis

The statistical analyses were carried out using SigmaPlot 12.5 (Systat Software Inc., Richmond, CA, USA) with the level of significance set at *p* < 0.05. Regional brain CCO activity was analyzed using two-way ANOVA (rearing condition × sex in each brain region), with post hoc analysis by Tukey HSD multiple comparison tests.

## 3. Results

### 3.1. CCO Activity

#### 3.1.1. Main Effects of Sex and Rearing

Females showed greater CCO activity than males in the dorsal striatum [F(1,37) = 8.121; *p* < 0.001] and the core of the nucleus accumbens [F(1,37) = 70.180; *p* < 0.001]. Likewise, females showed greater CCO activity in the CA1 field [F(1,37) = 31.403; *p* < 0.001] and CA3 field [F(1,37) = 10,660; *p* = 0.003] of the dorsal hippocampus, the CA1 field [F(1,39) = 161.238; *p* < 0.001] CA3 field [F(1,38) = 212.947; *p* < 0.001] and the dentate gyrus [F(1,37) = 68.191; *p* < 0.001] of the ventral hippocampus (Table 1).

One brain region showed treatment differences independent of sex condition, with MS females showing lower CCO activity than control females in the CA3 field of the dorsal hippocampus [F(1,37) = 28.956; *p* < 0.001].

#### 3.1.2. Interaction between Sex and Rearing

As shown in Table 1, significant interactions between gonadal sex and rearing condition were found in the prefrontal cortical regions, dorsal dentate gyrus, and the shell of the nucleus accumbens. MS groups had significantly lower CCO activity as compared to control groups in all frontal cortical regions (*p* < 0.01) and dorsal dentate gyrus (*p* < 0.001). The female control group showed greater CCO activity than the MS female group in the shell of the nucleus accumbens (*p* < 0.001) while there were no significant differences in CCO activity between the rearing condition in males.

Moreover, post-hoc tests of significant interactions indicated that the female control group had significantly greater CCO activity in all frontal cortical regions, dorsal dentate gyrus, and the shell of the nucleus accumbens as compared to the control male group (*p* < 0.001). In addition, MS males had significantly lower CCO activity in the cingulate cortex and the shell of the nucleus accumbens as compared to the MS female group (*p* < 0.001).

### 3.2. Effect of Sex and Rearing on the Concentrations of Monoamines Levels and Turnover in Selected Brain Regions

**Figure 1:**
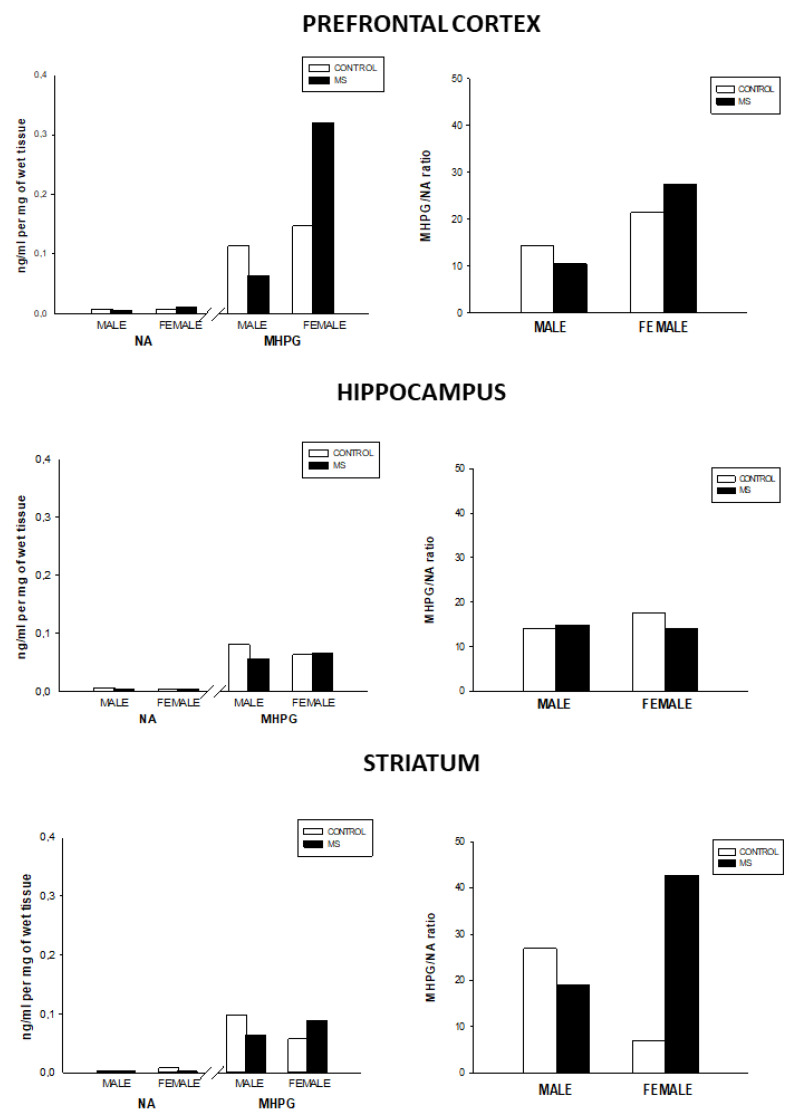
NA, MHPG levels (ng/mL per mg of wet tissue), and NA turnover (MHPG/NA) in the prefrontal cortex, hippocampus, and striatum for the control and MS groups.

Regarding the noradrenergic activity, the results shown in Figure 1 indicate that NA levels were not different between the experimental groups. However, MS females showed increased MHPG in the prefrontal cortex and norepinephrine turnover in the striatum as compared to the other groups. Conversely, MS males showed a slight decrease in MHPG in the prefrontal cortex. Likewise, MS males exhibited norepinephrine turnover decrease as compared to the control group.

**Figure 2:**
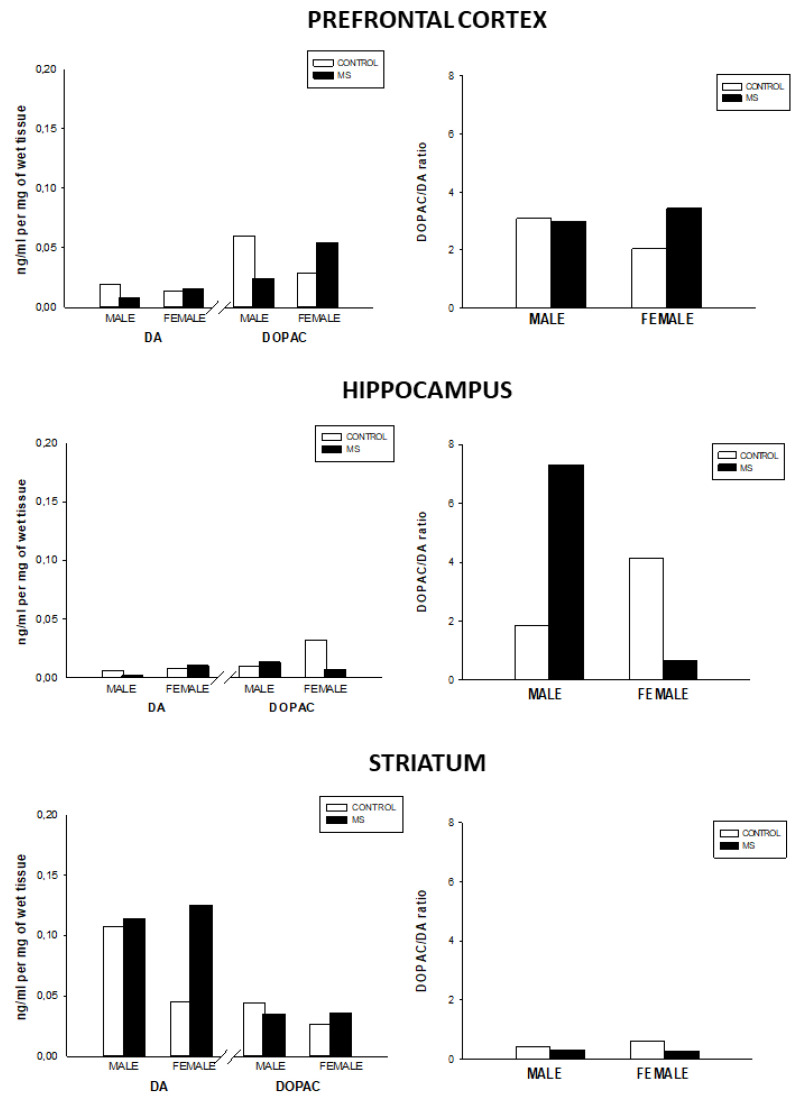
DA, DOPAC levels (ng/mL per mg of wet tissue), and DA turnover ((DOPAC/DA ratio) in the prefrontal cortex, hippocampus, and striatum for the control and MS groups.

Furthermore, female MS showed increased DA levels in the striatum as compared to the control group. Also, female MS rats showed increased DOPAC levels and DA turnover (DOPAC/DA ratio) in the prefrontal cortex (see Figure 2).

However, MS males had lower DOPAC in the prefrontal cortex as compared to the male control group. On the other hand, female MS rats showed increased dopamine turnover in the prefrontal cortex but decreased DA turnover (DOPAC/DA ratio) in the hippocampus. Conversely, MS males showed increased DA turnover in the hippocampus.

**Figure 3:**
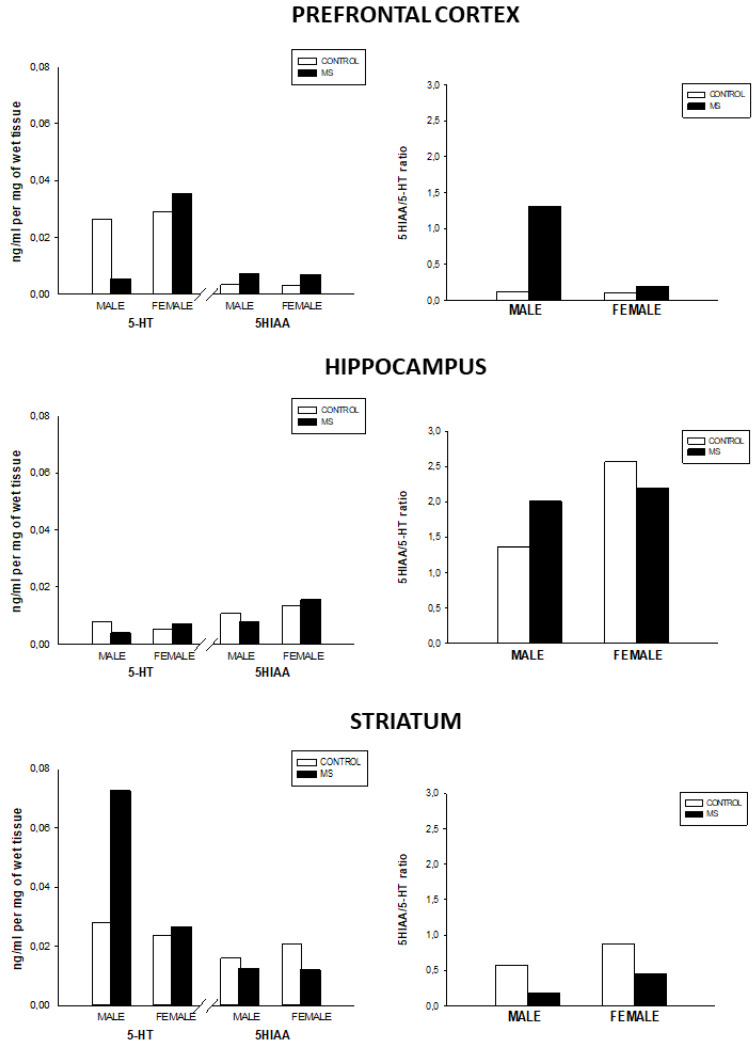
5-HT, 5-HIAA levels (ng/mL per mg of wet tissue), and 5-HT turnover (5-HIAA/5-HT ratio) in the prefrontal cortex, hippocampus, and striatum for the control and MS groups.

As compared with sex-matched control groups, MS males had lower 5-HT levels in the prefrontal cortex. However, male MS showed increased 5-HT levels in the striatum and 5-HT turnover in the prefrontal cortex and hippocampus. Lastly, MS groups showed slightly lower 5-HIAA and serotonin turnover in the striatum as compared to the control groups in both sexes (Figure 3).

### 3.3. Effect of Sex and Rearing on Cytokine mRNA Levels in Selected Brain Regions

**Figure 4:**
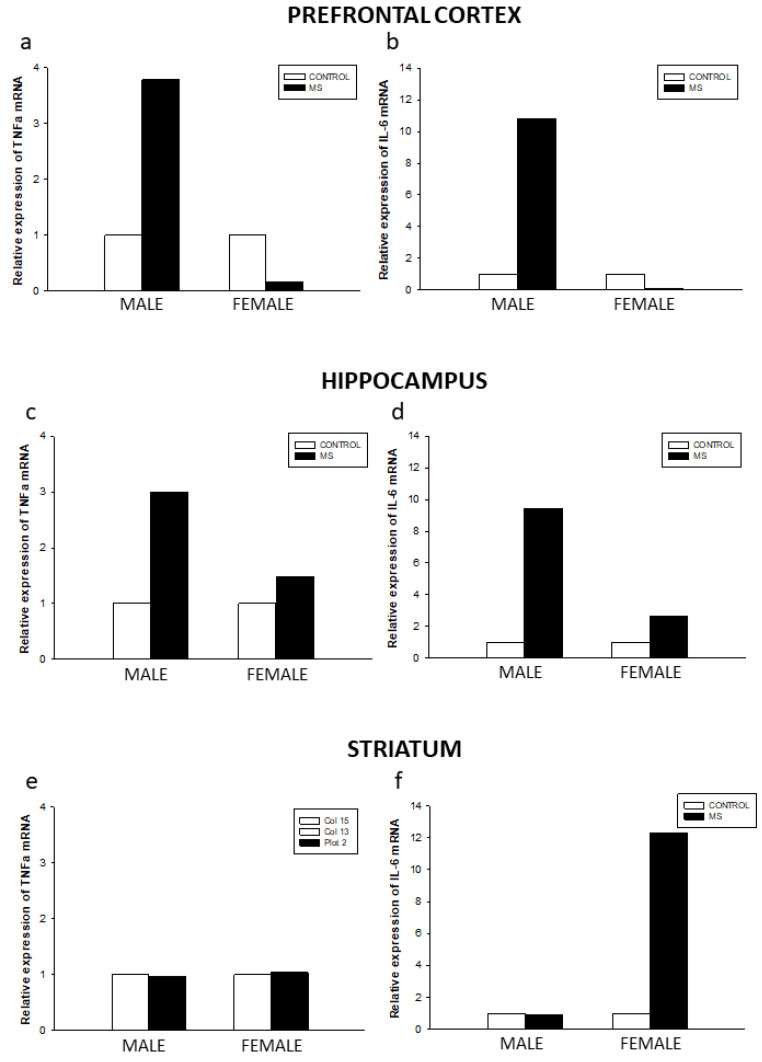
Effects of early maternal separation in IL-6 and TNF-α mRNA relative expression levels in the prefrontal cortex (**a**,**b**), hippocampus (**c**,**d**), and striatum (**e**,**f**) of male and female adult rats.

No differences were found between male and female control groups on the relative expression of IL-6 and TNF-α mRNA in the prefrontal cortex, the striatum, and the hippocampus (Figure 4).

The effects of MS were most pronounced on the relative expression of cytokine levels in males than females in the prefrontal cortex and the hippocampus. Moreover, IL-6 and TNF-α mRNA levels were greatly increased in the prefrontal cortex and the hippocampus of the male MS group as compared to the control group. Furthermore, IL-6 mRNA levels were considerably increased in the striatum of the MS female group as compared to its sex-matched control group.

## 4. Discussion

Early exposure to stress by prolonged MS clearly showed sex-dependent effects on adult rat brain mitochondrial function, neuroimmune response, and monoaminergic activity. Our findings are in line with a previous study reporting sex differences in brain oxidative metabolic capacity after a different MS paradigm of ELS in juvenile rats [32]. This study showed that a shorter MS protocol (PND 2-6 and PND 9-13, 6 h/day) decreased CCO activity in the prefrontal cortex and nucleus accumbens in both female and male 14-day-old rats. These results fully agree with our study, because CCO activity was also significantly reduced in male and female MS groups in the prelimbic and infralimbic areas of the prefrontal cortex, and the nucleus accumbens shell. MS female rats also showed greater CCO activity reduction than males in the same brain regions. Likewise, a similar pattern of CCO activity sex differences was found in the cingulate cortex and the dorsal hippocampus. Decreased brain mitochondrial function (and CCO activity in particular) has been associated with exposure to chronic stress and anxiety in many experimental studies with animals [17,23,47]. However, the effects of ELS on the neuroendocrine response to stress critically depends on the timing and duration of the MS protocol. In particular, early MS during the two first weeks of life in rats seems to impair adult HPA axis response, because it overlaps with the ‘stress-hyporesponsive period’ (SHRP, PND 3-14 in rats) characterized by low ACTH and glucocorticoid levels in response to external stressors [7,32]. However, prolonged MS during the entire lactation period (PND 1-21) in rodents may have different effects on the HPA axis function during adulthood, as already reported regarding brain CCO activity [34,35]. Accordingly, increased brain CCO activity in MS female rats as compared to same-sex controls was reported using a short MS period (PND 1-10) that only partially overlapped the SHRP [35]. Therefore, the effects of prolonged MS during the entire weaning period seem to have detrimental effects on brain mitochondrial activity, as compared with shorter MS periods, probably by directly affecting normal HPA axis development.

In particular, low mitochondrial function, expressed as reduced mitochondrial respiratory capacity, decreased ATP levels, and increased oxidative stress in the nucleus accumbens shell has been reported in rats showing anxiety-like behavior [39,48,49,50]. Moreover, reduced CCO activity in the prefrontal cortex, the hippocampus, and the mesolimbic dopaminergic system (including the nucleus accumbens shell) have been reported after chronic social defeat in rodents and in congenitally-helpless rats showing depression-like behavior [43,47,51]. Accordingly, a recent pilot study in patients with major depression and major depressive episode showed decreased CCO activity in the prefrontal cortex inversely correlated with depression severity [52]. Moreover, the dorsal hippocampus was also affected by MS, since the dentate gyrus showed significantly lower CCO in both male and female rats. ELS by MS during PND 14-21 [53] and PND 1-21 [54] has been reported to particularly affect the normal anatomical and functional development of the hippocampus and prefrontal cortex in rodents. In this regard, impaired spatial learning and memory in male rats and deficits in behavioral flexibility in female rats have been associated with hippocampal and prefrontal cortex dysfunction after MS in rodents [35,37,55]. However, we found no significant effects of MS on brain CCO activity using a similar MS protocol in our previous study [34]. This discrepancy with our previous study could be due to multiple comparison errors and the small sample size used, since in this study, only males were analyzed and compared with additional groups reared under environmental enrichment conditions. In fact, CCO activity differences between MS and control male groups in this study were like those reported here in the same brain regions, but group differences almost reached statistical significance (*p* = 0.054).

The mechanisms underlying decreased brain CCO activity after exposure to early stress are currently speculative, although mitochondria have been proposed as key mediators in brain programming by exposure to ELS [56]. Mitochondrial bioenergetics, glucocorticoid, and neurosteroid synthesis and metabolism, and reactive oxygen species (ROS) production have been proposed as main mechanisms explaining the relationship between ELS and reduced mitochondrial function [16,18,20,56]. It is known that ELS can induce epigenetic changes in brain regions associated with the physiological stress response, and program hippocampal and HPA axis functions leading to increased stress responsivity in adulthood [1,56]. Prolonged increased energy demands in brain cell mitochondria driven by the stress response system (including enhanced HPA axis and sympathetic system functions) could cause increased reactive oxygen species (ROS) production that can overwhelm antioxidant cellular response [17,20]. ROS could damage mitochondrial DNA responsible for the synthesis of cellular respiration enzymes like CCO and, in turn, decrease CCO activity in brain cells. In addition, freely circulating immunogenic mitochondrial molecules in plasma-like mitochondrial DNA and derived proteins (N-formyl peptides, also known as ‘danger-associated molecular patterns’ or DAPs) released after mitochondrial damage could be recognized as foreign molecules and trigger inflammatory immune responses like cytokines released by leukocytes and microglia [17]. Mitochondria, in turn, regulate neurotransmission and physiological stress response and could induce abnormal brain function and behavior, since chronic neuroinflammation is associated with several mental disorders like depression and anxiety, among others [10,17,18,20,57]. In this regard, mitochondrial function impairment has been reported in human studies of several mental disorders like depression, anxiety, and schizophrenia associated with a history of ELS during childhood [20].

Interestingly, sexual differences have been reported in the diagnoses of anxiety and depressive disorders that peaks during adolescence and early adulthood, with women showing twice the lifetime rates of depression and most anxiety disorders [58]. Data from experimental studies in rodents have shown sex differences in HPA axis regulation after ELS and chronic stress [14,29]. Dysregulation of HPA function during early development has been suggested to increase adult vulnerability to depression and anxiety disorders [59,60,61,62]. In our study, female rats showed greater CCO activity in most of the selected brain regions as compared to males, and they also showed greater reduction of CCO activity after MS as compared to males. The organizational and activational effects of gonadal or sex steroids on developing HPA could be responsible for the sex differences reported here. Like glucocorticoids, androgens and estrogens regulate HPA axis function in opposing ways by directly acting on their components, thereby modulating the development of stress reactivity [63,64]. Estrogens can act via alpha estrogen receptors after chronic stress to inhibit local GABAergic neurons of the paraventricular hypothalamic nucleus, and therefore enhance HPA axis response to stress [63]. However, androgens inhibit HPA axis response by acting on androgen receptors on the parvocellular cells of the paraventricular nucleus, but it should be considered that androgens can be converted to estradiol by brain cells [63,64]. In particular, basal and stress-induced HPA function seems to be higher in female than in male rodents [63,65]. Moreover, sex differences in mitochondrial function and biogenesis by the direct influence of gonadal steroids on estrogen receptors could also be responsible for the sex differences in brain CCO found in control and MS rats [26]. However, contrary to our initial hypothesis, the CCO activity results would only partly support the higher vulnerability of females to the detrimental psychological and physiological consequences of chronic stress. Despite the potential neuroprotective effects of estrogens due to its antioxidant and anti-inflammatory properties on mitochondrial function, MS females showed decreased CCO activity in many brain regions as compared with control females. Probably, the more prolonged and greater response of the HPA axis to ELS of female rats compared to males could cause long-lasting detrimental effects on mitochondrial brain function. Therefore, chronic glucocorticoid exposure during the first weeks of life in females could decrease the activity of CCO and/or increase mitochondrial reactive oxygen species (ROS) by acting on glucocorticoid-response elements that regulate mitochondrial DNA gene expression [16]. In this regard, the low levels of circulating estrogens before puberty in female rats would not have prevented the detrimental effects of ELS by MS during the first weeks of life.

On the other hand, sex differences were found in monoaminergic activity after MS. As compared with sex-matched control groups, MS males showed lower 5-HT levels and increased 5-HT turnover in the prefrontal cortex and the hippocampus, and increased dopamine turnover in the hippocampus. Conversely, norepinephrine turnover decreased, and 5-HT levels increased in the striatum of MS males. Female MS rats showed increased dopamine turnover in the prefrontal cortex, but decreased dopamine turnover in the hippocampus. In addition, female MS rats also showed increased norepinephrine turnover in the striatum. Sex differences in brain monoamine levels in similar regions have been reported in rodents after MS [13,14,66,67,68]. Our results only partially agree with previous studies, probably due to the different MS protocols available, that mainly differ in the time length and age when MS was applied. In this regard, our results agree with previous studies reporting decreased 5-HT levels and increased 5-HT turnover in the prefrontal cortex and hippocampus of male rats after prolonged MS from PND 2-21 [38,68] and short MS from PND 2-14 [69].

5-HT is a key neurotransmitter for typical brain development involved in a wide set of brain functions, including emotional and cognitive processes altered in adults by early exposure to MS [12,70]. Deficits in 5-HT levels of the hippocampus and prefrontal cortex in males after short (PND 1-14) and prolonged (PND 2-21) MS could be related to anxiety-like and depression-like behaviors, impulsivity or behavioral disinhibition, and impaired stress response as already reported [13,71,72]. These 5-HT deficits agree with decreased CCO activity found in the same brain regions of MS males, a result that suggests that males are also affected by MS, but probably showing a different behavioral response profile as compared with females. For example, female rats are less likely to show impulsive behavior as compared to males after MS, as related to impaired development of the prefrontal cortex [41]. Dopamine turnover in the hippocampus and norepinephrine turnover in the striatum showed opposite changes in males and females after MS. Similar changes in catecholamine levels in the hippocampus and striatum have been found after chronic ethanol administration in MS male and female mice [14]. Indeed, MS males in this study were more susceptible to the addictive effects of chronic ethanol intake and its adverse effects on the HPA axis stress response [14]. These sex differences in brain neurotransmitter levels and behavior could also be related to the sex-dependent disruption by ELS of the maturation of cortico-limbic circuits, as recently reported in a neuroimaging study in rats [73].

Lastly, MS induced a proinflammatory state in all the selected brain regions but showed a clear-cut sex difference. MS males showed increased IL-6 and TNF-α mRNA levels in the prefrontal cortex and the hippocampus, whereas MS females showed decreased TNF-α mRNA levels in the prefrontal cortex and highly elevated IL-6 mRNA levels in the striatum. These results also partially agree with studies mostly reporting long-term increased inflammatory response in similar brain regions after short MS during PND 1-14 [10,29,33,74,75]. It is well known that the HPA axis and the immune system are reciprocally regulated systems [10,61]. Sustained increased corticosterone or cortisol levels have been associated with an increased inflammatory response in the brain, modulated by monoamines like norepinephrine and 5-HT [10,76]. There is clinical and experimental evidence that the behavioral consequences of chronic stress and several mood disorders like depression are linked to increased neuroinflammation [61].

Our results suggest that males and females are also differentially affected by exposure to ELS, with MS males showing more brain regions with high levels of cytokines as compared with MS females. The few studies examining sex differences in neuroinflammation after chronic stress indicate that males are more affected than females [29,77]. Consistent with our findings, two studies reported increased cytokine levels and activated microglia in the hippocampus and prefrontal cortex of male rats, but not in female rats [78,79]. The protective effect of estrogens and progesterone in females with their known anti-inflammatory effects could partially explain our results regarding brain cytokine expression [29,80]. Several potential mechanisms have been suggested explaining the anti-inflammatory effects of estrogens and progesterone on brain cells. Estrogens and progesterone probably act on brain microglial cells by alpha- and beta- estrogen receptors and progesterone nuclear and membrane receptors in these cells and attenuate the microglia activation and cytokine release by these cells. In addition, estrogens and progesterone inhibit the induction of inflammatory mediators like inducible nitric oxide synthase, among additional potential mechanisms [29,80]. However, IL-6 levels increased after MS in the striatum of female rats, a result suggesting that female rats were also affected by MS. In this regard, depressive-like behavior was associated with increased IL-6 levels in the rodent brain [81]. Therefore, females also showed a more limited pro-inflammatory response as compared with males after MS. Our results are in line with recent findings supporting a sexually dimorphic response to chronic stress in mice, with males showing greater anxiety- and depression-like behavior, together with higher levels of pro-inflammatory cytokines and lower levels of brain monoamines, and females showing greater HPA axis activation, but less anxiety- and depression-like behavior [82].

## 5. Conclusions

In summary, ELS by MS caused a wide range of complex and sexually dimorphic effects on local brain energy metabolism, monoaminergic activity, and neuroinflammatory response. Prolonged MS decreased mitochondrial activity, altered dopamine turnover in the prefrontal cortex and the hippocampus, and increased norepinephrine turnover and IL-6 levels in the striatum of female rats. However, prolonged MS in males increased 5-HT turnover in the prefrontal cortex and the hippocampus, decreased dopamine turnover in the hippocampus, and increased TNF and IL-6 levels in the prefrontal cortex and the hippocampus. Our findings support the hypothesis about the neurobiological basis underlying the different adaptive responses between sexes to ELS, and their behavioral consequences in the adult stage [29,79]. However, further studies should address the complex neurobiological mechanisms involved in the sexual differences reported here. Our results underscore the urgent need for more studies, including female experimental animals, to evaluate sex differences in preclinical models required for translational research and human clinical trials.

## Figures and Tables

**Table 1:** Sex-specific effects of early life stress in regional brain CCO activity of adult rats. Data are expressed as mean ± standard error.

	Control Group		Maternal Separation Group		Effects (*P*=)		
	Male	Female	Male	Female	Sex	Separation	Sex × Rearing
Cingulate cortex (Cgl)	31.3 ± 1.1	41.7 ±1.0	26.7 ± 0.9	29.7 ± 0.9	<0.001	<0.001	**<0.001**
Prelimbic cortex (PrL)	32.1 ± 0.9	40.3 ± 0.8	27.2 ± 0.9	28.9 ± 0.7	<0.001	<0.001	**0.001**
Infralimbic cortex (IL)	30.6 ± 1.9	43.1 ± 0.6	25.8 ± 1.8	28.4 ± 0.5	<0.001	<0.001	**<0.002**
Doral striatum (dCPu)	27.3 ± 0.6	30.2 ± 0.4	29.3 ± 1.7	32.5 ± 0.6	**<0.007**	0.055	0.902
Accumbens nucleus, core (AcbC)	33.5 ± 1.4	42.4 ± 0.5	33.9 ± 1.3	43.7 ± 0.7	**<0.001**	0.430	0.689
Accumbens nucleaus, shell (AcbSh)	38.8 ± 0.5	52.1 ± 0.6	36.7 ± 0.9	45.1 ± 09	<0.001	<0.091	**<0.007**
Dorsal field CA1 of the hippocampus (dCA1)	22.1 ± 0.4	26.7 ± 0.7	22.2 ± 0.7	24.5 ± 0.2	<0.001	<0.091	0.076
Dorsal field CA3 of the hippocampus (dCA3)	24.7 ± 0.9	29. 4 ± 0.7	21.7 ± 09	22.8 ± 0.8	**0.003**	**<0.001**	0.504
Dorsal dentate gyrus (dDG)	32.4 ± 0.5	40.9 ± 1.3	27.0± 0.9	27.3 ±1.1	<0.001	<0.001	**<0.001**
Ventral field CA1of the hippocampus (vCA1)	23.7 ± 0.2	31.6 ± 1.0	22.2 ± 0.6	32.2± 0.6	**<0.001**	0.488	0.142
Ventral field CA3 of the hippocampus (vCA3)	22.8 ± 0.9	33.5 ± 1.1	20.2 ± 0.8	40.7 ± 1.4	**<0.001**	0.998	0.101
Ventral dentare gyrus (vDG)	24.05 ± 0.4	30.9 ± 1.2	21.6 ± 0.8	30.1 ± 1.	**<0.001**	0.054	0.247
